# Breast cancer services in Vietnam: a scoping review

**DOI:** 10.1080/16549716.2018.1435344

**Published:** 2018-02-23

**Authors:** Chris Jenkins, Luu Ngoc Minh, Tran Tuan Anh, Tran Thu Ngan, Ngo Tri Tuan, Kim Bao Giang, Luu Ngoc Hoat, Lynne Lohfeld, Michael Donnelly, Hoang Van Minh, Liam Murray

**Affiliations:** ^a^ Centre for Public Health, Queen’s University, Belfast, UK; ^b^ Institute of Preventive Medicine and Public Health, Hanoi Medical University, Hanoi, Vietnam; ^c^ Centre for Population Health Sciences, Hanoi University of Public Health, Hanoi, Vietnam; ^d^ UKCRC Centre of Excellence for Public Health, Belfast, UK

**Keywords:** breast cancer, cancer, Vietnam, health systems, scoping review, NCDs

## Abstract

**Background**: Breast cancer incidence has been increasing consistently in Vietnam. Thus far, there have been no analytical reviews of research produced within this area.

**Objectives**: We sought to analyse the nature andextent of empirical studies about breast cancer in Vietnam, identifying areas for future research and systemsstrengthening.

**Methods**: We undertook a scoping study using a five-stage framework to review published and grey literature in English and Vietnamese on breast cancer detection, diagnosis and treatment. We focused specifically on research discussing the health system and service provision.

**Results**: Our results show that breast cancer screening is limited, with no permanent or integrated national screening activities. There is a lack of information on screening processes and on the integration of screening services with other areas of the health system. Treatment is largely centralised, and across all services there is a lack of evaluation and data collection that would be informative for recommendations seeking to improve accessibility and quality of breast cancer services.

**Conclusions**: This paper is the first scoping review of breast cancer services in Vietnam. It outlines areas for future focus for policy makers and researchers with the objective of strengthening service provision to women with breast cancer across the country while also providing a methodological example for how to conduct a collaborative scoping review.

## Background

Globally, breast cancer is the most commonly diagnosed cancer in women, with over 1 million cases diagnosed annually [1]. In Vietnam, breast cancer incidence has more than doubled over the last two decades from an age-standardised rate of 13.8 per 100,000 women in 2000 to 29.9 per 100,000 women in 2010. This trend translates to an estimated 12,533 new (reported) breast cancer cases per year across the country [2].

In comparison with higher income contexts, incidence remains quite low. In the UK there were 55,222 new cases recorded in 2014 [3], while across Europe there is an age-standardised rate of 94.2 per 100,000 women [4]. Estimates from the International Agency of Cancer Registries (IARC) additionally indicated a decline in the breast cancer age-standardised incidence rate in Vietnam to 23.0 per 100,000 in 2012 (or 11,067 new cases). Despite this possible reduction, however, breast cancer still accounts for over 20% of cancers in women in Vietnam [5], and there is an absence of national data on survival and mortality. In light of these epidemiological trends, we collaboratively conducted a scoping review of available literature focusing on breast cancer services in Vietnam.

**Objectives of the scoping review**:

- Review all available published and grey litera-ture on the organisation of breast cancer services in Vietnam
- Chart data related to detection, diagnosis and treatment
- Identify themes relevant to systems strength-ening
- Identify areas for future research

## Methods

Our scoping review followed the five-stage framework developed by Arksey and O’Malley [6]: identifying the research or review question(s); identifying relevant studies; selecting the studies for review; charting the data from them; and collating, summarising and reporting results. According to Mays et al. [7], scoping reviews ‘can be undertaken as standalone projects in their own right, especially where an area is complex or has not been reviewed comprehensively before [and in order] to map the key concepts underpinning a research area and the main sources and types of evidence available’.

A scoping review of published and grey literature was deemed the most apt methodology for this project due to the lack of analytical reviews on breast cancer services in Vietnam. We identified and charted common themes and gaps regarding the detection, diagnosis and treatment of breast cancer. Our focus related to health system organisation, service availability and readiness, technology, human resources, financing and patient management as identified by the WHO Health Systems Framework [8].

In March 2017, we systematically searched for literature published in English in the MEDLINE, Web of Science, and JSTOR databases respectively since 2002 (when the Vietnamese National Cancer Control Strategy was published) using the keywords ‘Vietnam’ AND ‘Breast Cancer’. Eligible articles were papers and publications that reported empirical data about breast cancer detection, diagnosis and/or treatment services including articles about cancer in Vietnam that referred to breast cancer and breast cancer services (initial searches indicated that few studies examined breast cancer in isolation). The systematic search indicated clearly that no review on this topic has been published.

In April 2017, we then searched the electronic databases at the Hanoi Medical University Library, the Hanoi University of Public Health Library, the Vietnam Oncology Journal (K Hospital) and the Institute for Preventive Medicine and Public Health Library in Vietnam using the keyword equivalent of ‘Breast Cancer’ in Vietnamese and the same inclusion criteria noted above. We charted and synthesised the data in Vietnamese before translating the results into English and combining them with our initial findings.

We identified grey literature through an online search. We searched the websites of Vietnamese governmental ministries, international organisations and non-governmental organisations, sourcing government reports and guidelines, strategic and operational-related documents, organisational reports and statistics. We applied the same inclusion criteria as noted above and used in earlier steps.

The final step in our methodological approach involved ‘charting’ all eligible documents in terms of the organisational categories of screening and detection, diagnosis and treatment. We extracted relevant details (including contextual information such as socio-economic data, demographics and incidence rates) and charted them to assist with our analysis of the findings. We consulted international guidelines from the WHO [9], the Breast Health Global Initiative (BHGI) [10], the European Society for Medical Oncology [11], the Asian Oncology Summit [12] and key articles in journals on global surgery and cancer control in low- and middle-income countries [13,14]. We compared recommendations from the guidelines with the results from our review, and by identifying discrepancies and gaps we sought to make recommendations for future areas of focus to strengthen Vietnam’s response to breast cancer.

## Results

Six articles and two abstracts that were published in English and five journal articles that were published in Vietnamese met our criteria and were included in the review. We included a further seven articles that focused specifically on risk factors for breast cancer in order to provide contextual and demographic information including articles on NCDs, global surgery and general cancer control in Vietnam. A search and review of grey literature produced eight documents comprising reports from government, public agencies and non-governmental organisations (see Flowchart 1 and Table 1).

**Flowchart 1.**
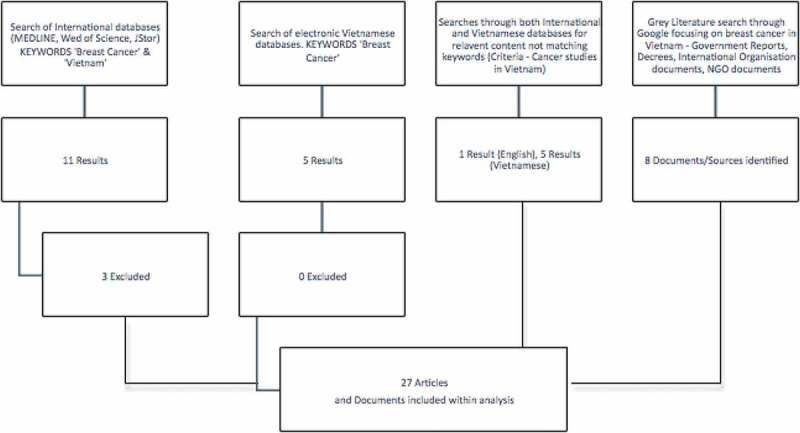
A flow-chart showing the process of article selection for this review.

**Table 1.** Breast cancer in Vietnam: articles identified within the scoping review (not inclusive of grey literature, e.g. government reports/decrees/NGO reports).

Author	Date	Title	Findings	Language
Meeting inclusion criteria (keywords: ‘Breast Cancer’ & ‘Vietnam’ (English) and ‘Breast Cancer’ (Vietnamese) in the title)				
Trieu, P. D. et al.	2017	Risk factors of female breast cancer in Vietnam: a case-control study	● Significant association between breast density, menopause status, number of pregnancies, number of babies born, hormone use and levels of physical activity with breast cancer in Vietnamese women	English
Nguyen, J. et al.	2016	A matched case-control study of risk factors for breast cancer risk in Vietnam	● Body mass index, lower parity, and later first childbirth all associated with breast cancer	English
			● Four or more births significantly reduced the chance of breast cancer	
			● No significant difference between breast cancer and age at menarche, age at first parity, total months breastfeeding, oral contraceptive use, and menopause	
Trieu, P. D. Y. et al.	2015	Female breast cancer in Vietnam: a comparison across Asian specific regions	● Breast cancer is the most common cancer for women in Vietnam. In the 1990s, the most common site-cancer was cervical/uterus	English
			● Breast cancer is commonly diagnosed at later stages (Stage II = 61.2%) and in women between the ages of 45 and 55	
			● Geographical variations. Hanoi has almost double the incidence of Ho Chi Minh City	
Le Thanh Duc et al	2015	The role, efficacy, feasibility of neoadjuvant AP regimen in inoperable stage III breast cancer (PhD thesis)	● By using AP regimen as neoadjuvant chemotherapy, the survival of the patients with inoperable stage III breast cancer was improved	Vietnamese
Nguyen, T.T.C. et al.	2014	Cost–utility analysis of Trastuzumab in treatment of metastatic HER2-positive breast cancer in Vietnam	● The treatment of metastatic HER2-positive breast cancer with Trastuzumab is considered cost-effective in Vietnam	English
Dieu, B.	2013	Trends of breast cancer in Vietnam	● From 2004 to 2008, the prevalence of breast cancer increased rapidly from the age of 30 to 34 and peaked at 55–59 at the rate of 135.0/100,000 people	Vietnamese
Lan, N. H. et al.	2013	Survival probability and prognostic factors for breast cancer patients in Vietnam	● Survival rates for breast cancer in Vietnam is lower than other countries of similar socio-economic level and that have similar stages of diagnosis	English
			● Married women with breast cancer have a significantly lower survival rate than unmarried women, and women diagnosed at later stages had worse survival rates	
Lan, N. H. et al.	2013	Cost of treatment for breast cancer in central Vietnam	● Direct medical costs for the treatment of breast cancer in Vietnam were estimated at $975 per patient (range: $11.7–3955)	English
			● Patients without health insurance had significantly lower levels of service utilisation	
			● Costs do not differ for patients diagnosed early or late, but survival times differ significantly	
Lan, N. H. et al.	2013	Cost-effectiveness analysis of a screening program for breast cancer in Vietnam	● Implementation of a CBE programme for women aged 40–55 would increase life years gained	English
			● CBE screening in Vietnam, by WHO criteria, is considered a cost-effective screening intervention	
Thuan Tran Van et al	2013	Breast cancer and risk factors related to family history in Vietnamese women	● Approximately 10% of breast cancers are inherited ● The rate of breast cancer is higher in those who smoke, drink alcohol, live in rural areas and have higher BMI, but the difference was not significant	Vietnamese
Dinh Nguyen, T.	2011	Breast cancer in surgery in Vietnam	● Breast cancer is often diagnosed late in Vietnam	English
			● Most women undergo mastectomy	
			● Demonstrated effectiveness in curative treatments, with low recurrence and high five-year survival	
Tu Nguyen Thi Nhu et al	2010	Factors related to breast cancer knowledge of women in Binh Dinh province in 2010	● General knowledge and awareness of symptoms and signs of breast cancer was low	Vietnamese
Duc Nguyen Ba et al	2003	Breast cancer	● Women who have mothers, sisters and daughters with breast cancer were at higher risk than those who have no family history of breast cancer	Vietnamese
Additional published articles included within analysis (keywords not in title)				
Thuan, T. V. et al.	2016	Cancer control in Vietnam. Where are we?	● 49.5% of women diagnosed at Stages III & IV	English
			● Screening campaigns are costly, and emphasis should be put on increasing levels of awareness of symptoms and treatment options	
			● High costs for patients and partial insurance coverage leads to high levels of treatment non-completion	
Tiep Do Quoc	2015	Knowledge of cancer prevention in Quang Binh province	● Knowledge about the risk of breast cancer was limited	Vietnamese
			● Knowledge about signs or symptoms of breast cancer was also low	
Thuan Tran Van	2013	Cancer prevention	● Increased risk of breast cancer in women with menstruation after 18 years of age; the first births over 20 years old are statistically significant	Vietnamese
Dieu, B. et al.	2012	Cancer challenges and national cancer control programs to 2020 (Vietnam)	● The breast cancer incidence in Vietnam has increased steadily over the last decade from a crude rate of 13.8 per 100,000 women in 2000 to 29.9 per 100,000 women in 2010, with an estimated 12,533 breast cancer cases in the country. The estimated number of breast cancer cases in 2020 is 38.1 per 100,000	Vietnamese
Duc Nguyen Ba et al.	2010	Results of national cancer program 2008–2010	● 9 of 63 provincial hospitals had no functioning pathology departments and 10 cannot admit patients with cancer. Cancer patients are referred to other hospitals with oncology departments	Vietnamese
Vach Trinh Huu	2010	Assessment of the need for cancer prevention for the community in Hanoi, Hue and Ho Chi Minh City	● Only 16% are aware of the risk factor of non-breastfeeding. Other factors such as not having children or late first childbirth, unhealthy diet, smoking, using hormone therapies over a prolonged long time (including birth control pills) were very low	Vietnamese
*Grey Literature (Government Reports, NGO reports etc)*				
Vietnam Women’s Union	2017 (accessed)	Awareness raising for breast cancer in women in Vietnam (2013–2015)	● After 3 years of implementation, the project, ‘Awareness Raising for Breast Cancer in Women in Vietnam’ has been implemented in 180 communes	Vietnamese
We Care for Her Campaign	2017 (accessed)	We Care for Her campaign website	● Between 2013 and 2014, the campaign, ‘Breast Cancer at the age of 40’ conducted free breast screening for 4000 women in five provinces: Hanoi, Ho Chi Minh City, Da Nang, Thua Thien Hue and Can Tho	Vietnamese
			● In 2016, free screening was provided to 12,000 women over 40 years of age from October to November in Hanoi, Ho Chi Minh, Da Nang	
Ministry of Health, Vietnam	2015	Joint Annual Health Review 2014: Strengthening prevention and control of non-communicable disease	● Breast cancers diagnosed at early stages are less expensive to treat cancer diagnosed at later stages ● The main obstacles preventing scale up of screening include a shortage of trained personnel, lack of appropriate diagnostic equipment and the lack of health insurance reimbursement for screening services	English
				
			● With available capacity, the existing network of cancer prevention and control can only meet approximately 30–40% of the need for oncology services among the population	
Ministry of Health, Vietnam	2013	Decisions and guidelines for cancer diagnosis and treatment	● Surgical interventions should take place at national and provincial levels; Benign tumours can be surgically removed at district level.	Vietnamese
WHO	2011	Recommendations for strengthening NCD prevention and control in Vietnam	● Vietnam’s NCD services are vertically organised and disease-specific ● Among others, recommendations for improving NCD service provision include strengthening primary care services; developing effective monitoring and surveillance systems; and investing significantly in human resources to tackle NCDs	English
Harper, C. (WHO)	2011	Vietnam Noncommunicable Disease Prevention and Control Programme 2002–2010. Implementation review	● Details the establishment of the national target programme for NCD prevention and control ● ‘A key challenge is the organization of the health system in Vietnam’ in particular through hospital outpatient departments	English
			● Integrated, intersectoral approach is required to control and manage NCDs	
			● Establish a population-based model for breast cancer screening	
			● ‘Establish registries to follow up women who have been screened for breast cancer, to ensure treatment is provided’	
Vietnam Health Insurance Agency	2009	Decree 62/2009/ND-CP. Detailed regulations and implementation guidelines of articles related to the Vietnam Law on Health Insurance	● Cancer patients are reimbursed 80% of examination and treatment costs. 20% of costs are paid out-of-pocket. For certain drugs, only 50% of costs are reimbursed. These drugs include Trastuzumab and other specialist drugs for the treatment of cancers and breast cancer	Vietnamese
Vietnam Health Insurance Agency	2009	Intersectoral Circular No. 09/2009 on guidance on implementation of health insurance	● Provides an overview of demographic and economic categories related to the provision of health insurance	Vietnamese
				

### Screening and detection

Information was not broadly available for how breast cancer is detected in Vietnam. While some studies provide data on the numbers of women screened under various programmes there are very few data concerning how these programmes were organised and what methods were used. There is also very little information on patient management guidelines regarding, for example, patient referral for diagnostic confirmation and treatment after the detection of an abnormal lump or identification of other symptoms.

We found evidence of at least three large-scale localised, provincial and regional screening programmes that have been implemented in Vietnam. Between 2008 and 2015, the National Cancer Control Programme screened an estimated 100,000 women aged 30–54 for breast and cervical cancer [15]. The National Cancer Control Programme offers free screening for all women [clinical breast examination (CBE), ultrasound, and mammography in case of referral], but information was not found on either its broader coverage or strategies for scaling up existing programmes.

The Vietnamese Joint Annual Health Review is an annual report that focuses on one sector of the country’s health care system. The 2014 issue stated that over 120,000 women have been screened nationwide (predominately in Hanoi and Ho Chi Minh City) between 2008 and 2013 [16] and recommended ways to better prevent and control non-communicable diseases (NCDs), including cancer. The ‘Early detection of breast cancer and cervical cancer in women’ programme was also implemented in Hanoi between 2012 and 2014 by the Committee for the Advancement of Women in Hanoi, and reportedly screened 50,000 women. However, information was sparse about how these screening programmes were conducted. Furthermore, evaluations about their outcomes appeared to be absent, although the review noted ‘a shortage of trained personnel, (a) lack of appropriate diagnostic equipment, and the lack of health insurance reimbursement for screening services’ [16].

The ‘We Care For Her’ campaign additionally reported providing free breast screening for an estimated 4000 women across five provinces in 2013–2014. These services were expanded to reach 12,000 women in Hanoi, Da Nang and Ho Chi Minh City in 2015–2016 [17]. The We Care For Her campaign is implemented by the Cancer Patient Support Fund, Bright Future, and supported by the Swiss Embassy in Vietnam and the Roche Group.

Given the localised nature of these interventions, and the lack of systematic national screening, it has been estimated that less than 10% of eligible women nationally receive appropriate annual breast (and cervical) screening [18]. Screening activities are not covered under National Health Insurance [16], and furthermore, the majority of programmes report screening activities in large cities, perhaps exacerbating inequality in access between rural and urban areas. Vietnam remains a largely rural country, with an estimated 65% of its population (2016) still residing in rural areas [19].

Beyond a lack of systemic capacity, the number of women using screening and detection services may be related to low levels of public awareness and education about detection, symptoms, where to access services and how to conduct self-examination. Late diagnosis and low population awareness of symptoms were commonly noted reasons for late presentation by women with breast cancer [15,20,21]. Knowledge on the need for early diagnosis is relatively low. One study reported that 40.8% of 900 subjects cited knowledge on the need for early detection [22]. The Vietnam Women’s Union (who have representatives at every Commune (primary, local level) in Vietnam) has been active in running programmes to increase awareness about breast cancer and reported to have increased monthly breast self-examination among women across five provinces between 2013 and 2015 from 7.7% of surveyed women in 2013 to 88% in 2015 [23]. However, in-depth information was lacking on which specific elements of these programmes contributed to success.

A study was also completed on the cost-effectiveness of CBE as a screening method in Vietnam to reduce mortality and morbidity from breast cancer [24]. The authors describe how CBE has been the primary screening and detection method across six provinces where cancer registries were established in 2008. A costing simulation (Markov Model) showed that CBE screening and detection were very cost-effective when compared with the WHO criteria and guidelines, with the cost-effectiveness of the screening program estimated to be US$994.96 per life-year saved. More detailed information on CBE was, however, not available, such as how often CBEs were conducted by doctors (e.g. through opportunistic screening) or the level of training health staff had received on how to conduct a CBE.

### Diagnosis

The Ministry of Health of Vietnam developed guidelines for breast cancer diagnosis and treatment outlining location and facility type (level) where examinations, diagnostics and treatment are to be provided [25]. Diagnostic capacity has increased across the country, with the development of six specialist oncology hospitals (public sector) and 43 oncology units in general hospitals. However, a number of challenges for diagnostics remain. A survey of the 63 provincial hospitals in 2008–2010 found that nine (14%) hospitals had no functioning pathology departments, and pathological confirmation in other hospitals could be slow and at times inaccurate [26].

Six of the eight English-language articles reviewed highlighted that the overwhelming pattern is for women to be diagnosed at later stages and at younger ages than in European contexts. In one study of 129 eligible patients, 56.6% were diagnosed at Stage II, 27.1% at Stage III and 9.3% at Stage IV. About one-third (36.4%) of the women were diagnosed between the ages of 40–49 years, and another 32.6% between 50 and 59 years of age [27]. Similar results were shown in two articles based on the same data set of 1584 patients [21,28]. Later diagnosis was also predominant in another study where 64.2% of 4715 new breast cancer cases diagnosed in five provinces were found at Stage III or stage IV [29]. Two further papers, including one retrospective study on stages of diagnosis across five hospitals in 2009, found that 49.5% of the women were diagnosed with Stage III or IV breast cancer [15], while 67.7% in the second study were diagnosed at Stage II [30].

Stage of diagnosis strongly correlated with treatment outcomes, with women diagnosed at earlier stages having better survival rates and fewer complications from treatment. In one study, although no significant difference was found for cost of treatment between those diagnosed at an early versus late stage of disease, the survival times were significantly longer for those women diagnosed at an earlier stage [27].

Specific information on how breast cancer was defined across studies is limited and can be an important reason for variation in study results. In Vietnam, the main means of detecting breast cancer cited in the literature include ultrasound, hemotogram, CA 15.3, tumour biopsy or cytological tests [27] with some women having mammography and oestrogen-receptor tests, progesterone receptor tests and Her 2-Neu tests.

### Treatment

Treatment for breast cancer in Vietnam is provided largely at the national and provincial levels. Two national level hospitals, one in Hanoi (K Hospital) and the other in Ho Chi Minh City (HCM Cancer Hospital), provide the majority of specialised cancer treatment. The Ministry of Health issued a document in 2013 [25] that mandates that most surgical interventions should take place at provincial and national level facilities, with benign tumours treated at district-level facilities.

Surgery, either stand-alone or in combination with other treatment modalities, represented the most common form of treatment for breast cancer in the studies examined. In one study of 3684 breast cancer cases treated between 2004 and 2008, 94% of the patients underwent mastectomy [29]. This finding is echoed in other studies [27,28] that cite surgery (complete mastectomy or breast-conserving surgery) as the most common treatment, often in combination with chemotherapy, radiation therapy and hormone therapy. Preoperative chemotherapy has also become more widely used in Vietnam to reduce tumour size prior to surgery and breast-conserving surgery. This approach has been shown to reduce recurrence rates and increase the 5-year survival rate in Vietnam from 40% to 75% [31].

Another study revealed that 79% of the 948 women completing the study received surgery, while 10% received chemotherapy (primary treatment) [28]. Surgery was also the main modality used for 73% of the 636 patients lost to follow-up compared with 11% who received chemotherapy as their primary treatment. Hormone therapy was offered to 75% of the group finishing the study over the five years after receiving their primary treatment [28]. Another study reported that tamoxifen was the most common hormone therapy [27] although trastuzumab has also been considered as cost-effective in combination with standard treatment for metastatic HER2-positive breast cancer women in Vietnam [32].

Capacity at provincial hospitals greatly varies, however, with administrators in 10 of 63 (15.8%) of provincial hospitals surveyed between 2008 and 2010 stating they could not admit and treat patients for cancer because they lacked the necessary radiotherapy equipment [26]. Treatment options across the country widely vary depending on geographic location, with a strong rural/urban difference [21], and while most provincial hospitals officially provide surgical services, as of 2008 (more recent information not found), 10 of them stated that they could not offer this service and regularly referred patients elsewhere [33].

Two studies reported a high rate of treatment non-completion, as well as a large proportion of women who received no treatment. In one study, 10.2% of eligible patients received no treatment after diagnosis [28]. Being married and frequent migration were two contributing factors, with marital status strongly linked to poor treatment success rates. Lack of health insurance was also cited as a possibly significant predictor of treatment dropout, with 26.2% uninsured women abandoning treatment compared to 5.9% of those with health insurance [27,28].

Health insurance coverage varies based on demographic information such as age, and on other factors such as employment status and income level. Those working in the informal sector (34% of the workforce aged over 15) are the most likely to not have health insurance [16]. In 2013 an estimated 31.5% of the population was not covered by health insurance [16]. Of the 68.5% who are covered by insurance, 26% of that proportion is considered poor or ‘near poor’ and are in receipt of State subsidies to support their premiums [16]. Sullivan et al. examined treatment discontinuation, financial catastrophe, and out-of-pocket costs in 4585 patients receiving surgical treatment in LMICs, including Vietnam, reporting that 73% of patients in Vietnam faced possible financial catastrophe due to costs related to receiving surgical treatment. Partial health insurance coverage, which meant women had enough coverage and financial resources to begin but not necessarily complete treatment, was attributed as a principal factor in understanding this high proportion [14].

Within the current Law on Health Insurance, cancer patients with insurance are reimbursed 80% of examination and treatment costs and 20% of costs are paid out-of-pocket. For certain drugs, however, only 50% of costs are reimbursed. These include trastuzumab, which can cost between 200 million and 800 million VND per year for individual treatment (US$10,000–40,000). Chemotherapy (Herceptin) is estimated to cost 500 million VND per year (US$22,000) (2013 figures) [16]. Having to meet co-payments can therefore be highly restrictive [34,35].

## Discussion

The scope and range of the articles in this review show that there is a relative lack of data related to detection, diagnostic and treatment services for breast cancer in Vietnam. Studies were localised, and projects (for example related to screening) tended to be local or regional. Despite significant increases in breast cancer incidence [15], there is only a modest body of literature to inform decisions about the planning and provision of cancer services. A number of articles provided useful case-study approaches, particularly around risk factors and breast cancer treatment. However, there is no extensive, whole-system research or large population-based studies that have mapped breast cancer services from detection to palliative care. More specifically, there is a need for research about how people access services, what barriers exist and the quality of breast cancer care.

There are very few studies and trials on detection of breast cancer. Despite Lan identifying that ‘an early detection strategy for breast cancer should be developed to improve life expectancy of women with breast cancer in Vietnam’ [24], no papers identified interventions to improve detection. While initiatives for screening have been implemented by Government and other agencies, few screening programmes have been evaluated. There is additionally a need for studies that test which characteristics of detection programmes and services help to improve engagement with the medical care system and downstage diagnosis.

There are knowledge gaps regarding the management of women with breast cancer related to where (and to whom) women present within the health system. Generally, there is a need to improve our understanding about how women with breast cancer seek help and interact with the health care system. Closing these knowledge gaps would contribute to research-informed planning and resource allocation. The results of the scoping review suggest that primary care is under-utilised and that patients tend to seek cancer services at a tertiary care level [36] and often when the cancer is at an advanced stage. The provision and delivery of information about the availability of breast cancer services in primary and community care would strengthen the health system overall. Our review showed that there has been no review of levels of awareness among health professionals about breast cancer symptoms in relation to whether health care professionals are able to conduct CBEs and appropriately refer women with abnormal lumps and other symptoms for diagnostic confirmation. A lack of information on human resources, capacity and areas for strengthening, was a key finding within this review.

Breast cancer treatment and health system guidelines have been developed for low- and middle-income countries [10,13,14]; and Vietnam developed guidelines for treatment options related to stage of diagnosis [28]. However, the nature and extent to which there is a good match between guideline recommendations, planned activities and service provision are not clear. The provision of good-quality, safe and timely surgery is the primary breast cancer treatment recommendation for countries with resource limitations [14,37]. It is noted that while increasing a country’s surgical capacity to treat cancers is expensive, it is a more realistic option (because it is more easily incorporated into existing health systems) than scaling up chemotherapy or radiotherapy services [13]. Mastectomy remains the most available, effective basic surgery option in low-income countries. In countries with radiotherapy capacity, breast-conserving surgery with radiotherapy is additionally recommended and is considered as effective as mastectomy [13].

In Vietnam, surgical treatment is used in most cases of breast cancer. No data were found to indicate clearly that up-to-date surgical guidelines were in place and whether or not systematic, robust evaluations were regularly undertaken. There is a need to give consideration to the role of clinical audits in the health system as ‘national surgical audits are a potent method for improving systems of cancer surgical care’ [14]. More data on the numbers of women receiving chemotherapy and radiotherapy would be useful, as would data on treatment outcomes. This would allow forward planning by placing such data in the context of evaluating the capacity of the system to treat women with chemotherapy and radiotherapy. Data on treatment outcomes would be useful within an audit, and as a measure of success within any evaluation.

The European Society for Medical Oncology recommends that breast cancer treatment should be carried out in ‘breast units’ by multidisciplinary teams of oncologists, surgeons, radiologists, pathologists and, where appropriate, reconstructive surgeons and psychologists [11]. While these conditions may not exist in low- and middle-income countries, they represent a benchmark of best practice. Where it is not realistic to create specific cancer units, it is recommended that cancer treatment should be organised and incorporated into the existing health system, with an integrative approach linking up services across healthcare levels [38]. Consideration should additionally be given for the creation of teams of professionals working at lower levels of the health system primarily with the responsibility of detection and referral. It is unclear to what extent this approach has been implemented in Vietnam.

‘Preoperative chemotherapy is the preferred primary treatment for locally advanced breast cancer because it allows an early assessment of sensitivity to treatment as well as breast conservation’ [10]. Given the high costs, radiotherapy and chemotherapy may not be considered realistic treatment options in many low- and middle-income countries. Where resources and facilities exist, it is recommended that they should be organised at the secondary level, making the service more accessible to patients [10]. Radiation therapies should be available, given their efficacy in treating early-stage breast cancer and as breast conservation treatment [10]. In Vietnam, there appears to be a low capacity to provide radiotherapy treatment. Studies should explore whether or not this is a cost-effective option for treatment in the future.

Guidelines suggest that tamoxifen should be used in hormone therapy for low- and middle-income settings [10]. Vietnam’s current practice seems to be in line with this recommendation. For oestrogen-receptor positive cancers, five years of endocrine drug therapy is recommended. This is realistic in low-resource settings using tamoxifen (generic). ‘Aromatase inhibitors produce better results than tamoxifen and are recommended for countries with enhanced and maximal resources, but cost restraints make tamoxifen a very reasonable alternative’ [10]. Trastuzumab is not generally considered a cost-effective option in low-resource settings [13], a conclusion that is contrary to one of the articles returned in our literature search [32].

WHO [9] recommended that cancer diagnostic and treatment plans (not just targets) should contain clear treatment pathways. These plans should include priority setting, quality and standards checks, information on the organisation of services, service integration and linkages to palliative care, and the establishment of clinical guidelines for treatment. While Vietnam has a National Cancer Control Plan, it is not clear whether it incorporates systematic planning, reporting and evaluation mechanisms. Vietnam does not have a full, comprehensive and operational cancer registry, which represents a limitation in terms of providing data to inform decisions. Only nine hospital-based cancer registries exist across the country [15] out of 63 provincial hospitals. This shortage of data is not unique to Vietnam, and represents a challenge across many low- and middle-income settings [39].

Data are also sparse regarding patient’s perceptions of social and cultural barriers in relation to access to, and use of, services. No qualitative studies have been conducted on breast cancer in Vietnam. Research is required on the social, economic and cultural determinants of breast cancer diagnosis, treatment and survival in Vietnam, particularly given the later detection rates among married women. It is likely that economic barriers such as the role of health insurance and the capacity to make co-payments play a role in late detection, but there is a need for empirical studies to investigate this issue. The cost analysis conducted by Lan et al. [27] did not account for direct non-medical costs (e.g. travel, accommodation and time) and indirect costs (lost income). A population-wide cost analysis and estimation is required, taking account for both direct and indirect costs of treatment services and accessing them. In the absence of research on social and cultural barriers to accessing diagnostic and treatment services, studies conducted in expatriate communities may provide insights useful for designing services. There is an extensive literature on health behaviours of Vietnamese communities living in the United States that may provide entry points for further research in Vietnam [40,41]. For example, research from Vietnamese women living in the United States shows that they are less likely to undergo CBE or mammography than American women [40]. While language barriers (and other barriers not relevant in a Vietnamese context) may partly explain these discrepancies, information about other barriers and perceptions may prove indicative and may help inform research in Vietnam.

Our scoping study also did not find any studies or grey literature that discussed palliation for breast cancer patients in depth. Systemic capacity appears to be lacking, especially given that as much as 70 percent of patients in oncology departments were estimated to be in late stages of cancer progression [16]. Very little information on palliation exists at the lower levels of the health system. Further study on this aspect of care and a fuller analysis into the capacity of the health system to respond to cancer are needed.

## Conclusion

There has been limited research published on breast cancer services in Vietnam. The information highlighted by this scoping study shows that in some areas, Vietnam’s cancer control and treatment strategies are in line with international recommendations for low- and middle-income countries (for example, in relation to surgery and hormone therapies), and in others, Vietnam does not meet international guidelines (for example, having a detailed cancer control plan, systems of registration and evaluation, and population-wide cancer registries).

The development of a national breast cancer control and treatment programme should be a key priority for the Ministry of Health, incorporating systematic and nationwide screening activities to downstage breast cancer diagnosis. The Ministry of Health has developed long-term goals, including the extension of screening programmes and building systemic capacity for diagnosis and treatment (both in facilities and in human resources) [16], but, as highlighted by Trieu et al., ‘In Vietnam, despite increased health care and public awareness, a breast cancer national control program as recommended by the WHO has not yet been established’ [21].

More research from the perspective of women with breast cancer is required, and clinicians and patients should be included in the planning of cancer control and treatment systems. Further work is required to support these processes, as well as further research investigating the different social and structural barriers that contribute to late diagnosis and treatment non-completion.

## Paper context

Given the lack of synthesised information on breast cancer in Vietnam, this review hopes to be of use to both policy-makers and researchers as they seek to strengthen breast cancer services in the country. The inclusion of information in Vietnamese makes available data to an international audience that would have been previously inaccessible. This review shows the need for further research dedicated to the strengthening of breast cancer services.
